# Pierre-Marie Gagey and the Evolution of Posturology: Unraveling the Complexity of the Fine Postural Control System

**DOI:** 10.7759/cureus.69052

**Published:** 2024-09-10

**Authors:** Orlando Conde-Vázquez, Sofía O Calvo-Moreno, Philippe Villeneuve

**Affiliations:** 1 Department of Functional Biology and Health Sciences, University of Vigo, Pontevedra, ESP; 2 Department of Physical Therapy, University of Camilo José Cela, Madrid, ESP; 3 Models, Dynamics, Corpus (MoDyCo UMR 7114), Paris Nanterre University, Nanterre, FRA

**Keywords:** historical vignette, muscle tonus, postural balance, posturology, stabilometry

## Abstract

The subtle sway of human standing posture began to be studied in the 19th century. Since then, numerous approaches - from the early statokinesiometers to the more advanced force platforms or 3D-posture recording systems - have been developed to understand human postural control physiology and its clinical implications. French physician Pierre-Marie Gagey made significant contributions to the field of posturology, a discipline focused on the intricacies of human postural control. From the 1950s, he advanced the field through observation and experimentation, particularly exploring the relationship between postural muscle tone and the fine postural control system. Gagey's collaboration with researchers of that time led to the refinement of stabilometry, a technique for measuring body sway in standing positions. Throughout his career, Gagey worked across multiple disciplines, including ophthalmology, podiatry, psychology, and the study of the vestibular and stomatognathic systems, to develop a comprehensive approach to postural assessment and treatment. His efforts culminated in the standardization of stabilometry, ensuring consistent and accurate measurements across different platforms. Gagey's work emphasized the complexity of postural control, highlighting the integration of peripheral and central nervous system inputs in maintaining balance. His research has left an enduring impact on the field of posturology, providing a framework for understanding and treating postural disorders. Pierre-Marie Gagey passed away in 2023, but his pioneering contributions continue to influence the study and clinical practice of human postural control. This narrative review aims to pay tribute to Dr. Gagey's career and research activity.

## Introduction and background

Pierre-Marie Gagey, a French medical doctor, was among the first to recognize the complexities of human standing postural control, contributing significantly to the emergence of a new scientific discipline known as "posturology" [1]. We can refer to posturology as the discipline that studies the function of the human orthostatic posture and the factors that affect it. It is therefore framed in the physiology of human motor control, where it acquires its importance given the "postural deficiency syndrome" described by Da Cunha [2] that leads to pain symptoms, imbalance sensations, ophthalmologic, and proprioceptive dysfunctions. From the ancient study of the postural sway described by Vierordt in 1860, which recorded these movements by attaching a pen to the tip of soldiers' helmet and drawing on a sheet of paper covered with charcoal [3], to the most elaborate stabilographic recordings of today, we will carry out a narrative review of Pierre-Marie Gagey's research and his contribution to this discipline. As he often remembered us, "a postural patient is a patient who has difficulty staying upright" [4].

As Gagey often explained, the idea first occurred to him in the 1950s while he was working as a medical doctor treating injured construction workers in the Paris region [4]. Dr. Gagey was puzzled by how these workers talked about dizziness or vertiginous sensations following mild cranioencephalic trauma (CET) presenting negative medical tests (otorhinolaryngological, otological, and neurological), even for prolonged periods of their lives. This curiosity marked the beginning of Dr. Gagey's investigation, which led him to connect with various researchers and delve deeper into the complex system of human postural control. Among them were Jean-Bernard Baron, research director at the French National Center for Scientific Research (CNRS), and ophthalmologist of the Saint-Anne Hospital in Paris; Henrique Martins da Cunha, Professor of Physical Medicine and Functional Reeducation at Lisbon University; and Dr. Tadashi Fukuda, Head of the Department of Otolaryngology at Gifu Medical School in Japan. Together, they identified postural muscle tone as a key factor in evaluating both normal and dysfunctional subjects in a standing position [5]. To further develop this new branch of knowledge, Gagey co-founded the French Association of Posturology (AFP) in March 1984.

Meanwhile, the Ukrainian V.S. Gurfinkel was already working somehow to represent the natural human oscillation in an upright position [6,7]. From the early statokinesiometers [8] till now, passing through the two-degree platform by Nashner [9], the scientific community finally accepted stabilometry as a reliable and valid tool to measure the body sway of human standing, under certain conditions [10-13]. Therefore, researchers finally had a way to record the small oscillations caused by the continuous postural adjustments of human standing against the force of gravity. In the 1980s, American researchers Anne Shumway-Cook and Fay Bahling Horak conveniently developed a series of tests to control the interaction between the three major postural management systems: the proprioceptive, vestibular, and visual systems [14]. By altering visual conditions (using a blindfold or a visual-conflict dome) and surface conditions (using a hard, flat surface or medium-density foam), they evaluated the neurological inputs and the central nervous system (CNS) integration, observing human balance under different conditions [15], the so-called Sensory Organization Test (SOT) [16]. Moreover, Gagey and Weber recognized that many individuals exhibited distinct posturographic behavior despite the absence of diagnosed neurological disorders, with many of these patients presenting similar complaints [4]. The key point is that clinicians knew well those conditions when the balance where out of the support polygon, but not when being within the normal limits of equilibrium, the patients showed clinically abnormal conditions. In other words, only small oscillations under 4° arc impact muscle postural tone, a phenomenon studied in posturology, which analyzes the “fine-tuned postural system” [4,17]. This narrative review aims to highlight Dr. Gagey's contributions to the field of human postural control systems.

## Review

Family history

Born on May 6, 1926, in Trier, Germany, Pierre-Marie Gagey grew up in Burgundy, France. His father was an artillery general, and his mother was a homemaker. The family was large, with 13 children. Gagey excelled in mathematics, one of his passions, at the Lycée Sainte-Geneviève (Ginette), a prestigious school known for its high success rate in preparing students for competitive examinations in major engineering and business schools. These mathematical skills later served him in the design and study of stabilometric parameters using force platforms [11,12]. He studied medicine in Dijon and Paris, graduating in the 1950s, and completed his medical thesis in 1954. He was ordained a priest by the Jesuits, but in 1970, he married Alix Fellot, a painter and mother of four children. Together, they adopted one more child.

The birth of posturology

**Figure 1:**
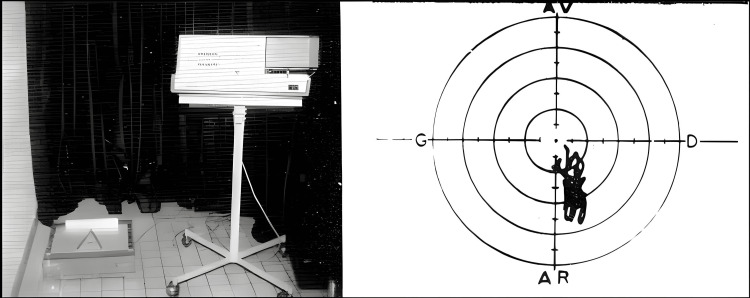
Left: Statokinesiometer in Baron's lab. Right: Statokinesiogram. The graph shows the course of the center of pressure (CoP) movement on the coronal and sagittal planes. Source: from the personal archives of P. Villeneuve (with the kind permission of the author).

Prompted by persistent doubts in the approach and treatment arising from clinical cases of cranial trauma, Gagey's pivotal meeting with Jean-Bernard Baron in 1972, whom he regarded as the true father of posturology, marked a significant turning point. This ophthalmologist, already working with a statokinesiometer (Figure 1), was one of the founders of the International Society of Posturography (ISP) in 1969, later renamed the International Society for Postural and Gait Research (ISPGR) in 1986. Today, the society includes more than 500 members across over 20 countries worldwide [18]. Dr. Baron investigated the impact of small excisions in the oculomotor muscles of fish and rats on the muscular tonus, causing a unilateral increase in paravertebral tone in these animals [19]. Together, they presented their research about the tonus variations of the tonic muscles related to oculomotor activity at the second ISP World Congress in Smolenice, Slovakia [20]. Along with Victor Gurfinkel, they honored J.B. Baron for being the first to guide them toward understanding the non-linear dynamics of the fine postural system [21].

As an occupational doctor, Gagey and colleagues observed post-concussion tonus variations in hundreds of mild head trauma cases [22], which revealed posturographic alterations regarding “normality” [23]. However, what did “normality” mean in posturographic terms? To statistically highlight abnormalities in these studies, Dr. Gagey needed a record of normal values. He and his colleagues dedicated themselves to this task with zeal and enthusiasm, pointing out the ideal conditions for constructing a stabilometric recording platform [11] and, together with other researchers, offering the first set of normalized data on 100 healthy subjects through the 1985 standards (*Normes 85*) [24]. His "playing field" was ready, the stabilometry allowed him to know the intricate details of the postural sway, checking its variations by stimulating the oculomotor, proprioceptive, and vestibular systems of the subjects analyzed. Finally, posturology was born [25].

The development

Gagey and colleagues continued their research at the Medical Institute of Posturology, in Paris, where they founded the Association for the Development and Application of Posturology (ADAP) in 1986. Furthermore, he held the presidency of the AFP for 12 years, until 1996. At that time, Dr. Gagey approached and deepened his knowledge with a true curiosity about other medical disciplines and human systems related to posturology:

Ophthalmology

in addition to working with J.B. Baron, he collaborated with Claudie Marucchi to develop methodologies for using prisms in lenses to induce postural changes [26-29]. Together with Françoise Zamfirescu, a co-author of his latest book [30], Gagey et al. studied the influence of binocular and monocular vision on postural control [31,32]. He and Bernard Weber believed that the body had to know the relative position of the different body elements with respect to each other, through what he called "endocaptors" [4]. In the eye, this information is derived from the neuromuscular spindles of the extraocular muscles, a line followed by Gagey’s colleagues also [33-37].

Stomatognathic System

The influence of this system on patients did not go unnoticed by Gagey, but at first time, he did not understand how it could influence standing, as “stomatognathic sensors have nothing to do with posture!” [38]. Nevertheless, he knew the ancient work of Jacques Meyer, who showed the influence of trigeminal afferences in postural tonic orthostatic regulation [39,40], and later he met Alfredo Marino, an Italian orthodontist who developed the “Alphs” together with Philippe Villeneuve, a French podiatrist and osteopath. The Alphs are little pieces of a photopolymerizable composite of two millimeters in diameter and approximately one-millimeter thickness, which placed on the vestibular face of the incisors affect stabilometric parameters [41]. Occlusion and swallowing were also found to influence stabilometric parameters [42-44].

Vestibular Inputs

Early research on the high threshold of angular acceleration perception of the semicircular canals [4], along with more recent studies [45] and Gagey et al.'s experimentation [46-49], led Pierre-Marie and colleagues to believe that the vestibular system did not have a preponderant action on the fine postural control system, at least in bipedal stance. Today, we know that vestibular inputs inform superior-inferior translation (such as gravity) at velocities as low as 1.7 cm/s [50].

Somatic Inputs

Dr. Gagey et al. were aware of the influence of foot baroreceptors and mechanical receptors in the musculoskeletal system (such as spindles and joint receptors) on human postural control [5,51,52]. However, he recognized the complexity of addressing somatic dysfunctions related to postural control, despite emphasizing the importance of free spine and ankle mobility. For this reason, he became involved with the work of various French osteopaths, such as Francis Peyralade, and physiotherapists, such as Éric Matheron [30,33,53]. J.P. Roll’s experiments further supported Gagey et al.'s theories, showing that spindle information and plantar stimulation can modify postural control [54-58]. The significance of Roll’s discoveries was acknowledged by Gagey and his collaborators after Roll died in 2018 [59].

Podiatry

The pressure information from the foot soles and the neuromuscular spindles of the deep muscles of the foot and the muscles of the leg compartments was considered by Gagey. Working closely with Philippe Villeneuve, they observed that heel height affects postural control [51,60], that low-thickness insoles can influence postural control [61-63], and that cutaneous afferents are particularly important in various populations, including dyslexic children [64], individuals with fibromyalgia [65,66], the elderly [67], and those with chronic low back pain [68]. Leporck and Villeneuve identified the so-called “plantar support irritative spines” (EIAP in French), small foot sole areas causing subthreshold nociceptive stimulations that lead to postural or balance modifications while standing [69], which can be treated with specific types of foot insoles [70-72].

Psychology

French psychiatrist A. Soulairac examined the postural strategies of psychiatric patients and their chemical treatments using posturography [73,74]. Gagey et al. also considered the influence of cognitive aspects in postural control, advocating for new normalized stabilometric data that consider patients with psychiatric disorders [75].

**Figure 2:**
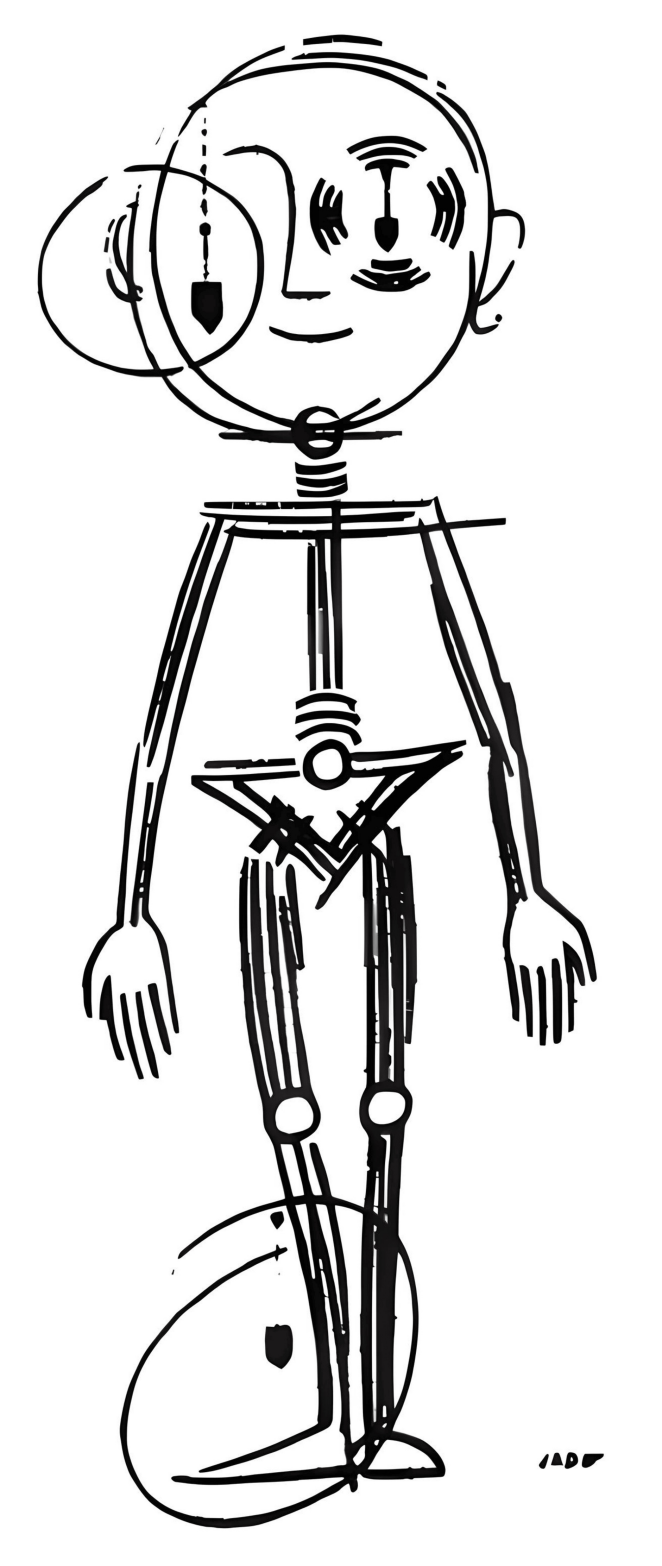
The Postural Man. The figure represents the main human entries for codifying gravity while standing. Extracted from [4], with the kind permission of Elsevier France through the Copyright Clearance Center.

In 1995, after a prolific career, he co-authored the main book on the discipline, along with Dr. Bernard Weber: *Posturologie : Régulation et Dérèglements de la Station Debout *[4]. It has served as a reference book for numerous clinicians interested in the subject and has been translated into several languages (Figure 2).

The clinical background

Dr. Gagey consistently emphasized the importance of posturology in restoring a patient’s postural health. To this end, he and colleagues sought to accurately substantiate the physiology of the fine postural control system, highlighting its integration within the CNS [17,38,76,77] and thoroughly evaluating its physiological foundations [78,79]. The significance of this endeavor was reflected in the journal published by AFP from 1984 to 1994, aptly titled "Critique of Posturology."

In addition, he and collaborators worked on developing or adapting testing sets for patient postural examinations [25,80] and created tools for postural re-adaptative training [81]. However, Gagey’s greatest contribution was in the field of stabilometry. First, he and colleagues claimed the minimum characteristics that a standardized force platform should possess, observing the position of the gauges, the type of material analyzed, and its digital characteristics among others [10,11]. Gagey and his colleagues studied various stabilometric parameters, derived from the movement of the center of pressure (CoP), as recorded by the force platform. Their initial studies focused on the X and Y axes, representing movements on the coronal and anteroposterior planes. By analyzing 100 healthy subjects, they provided standardized data for these parameters in 1985 [24], following guidelines previously proposed by the ISPGR [82]. These so-called *Normes 85* served as a reference for normalized data (especially in southwestern Europe), allowing the study of postural behavior in subjects outside the normal range, a task that Gagey undertook by studying thousands of subjects.

However, the initial technology available only allowed recordings at a frequency of 5 Hz, i.e., the device registered the CoP position five times per second. Gagey et al. noticed that orthostatic posture analysis adjusted to non-linear dynamics signal processing [83], thus needing more advanced force platforms that could record the CoP movement at 40 Hz [84]. Finally, Gagey and colleagues advocated for new standardization in stabilometry, including recording conditions [12,13,85]. Nevertheless, the reality today is that there is still no homogeneous standardization across force platform manufacturers, useful stabilometric parameters, or recording conditions [86]. Gagey and Weber opposed these inconsistencies throughout his life, arguing that homogenization was essential for posturology professionals to communicate effectively [87].

Posturology, paths ahead

**Figure 3:**
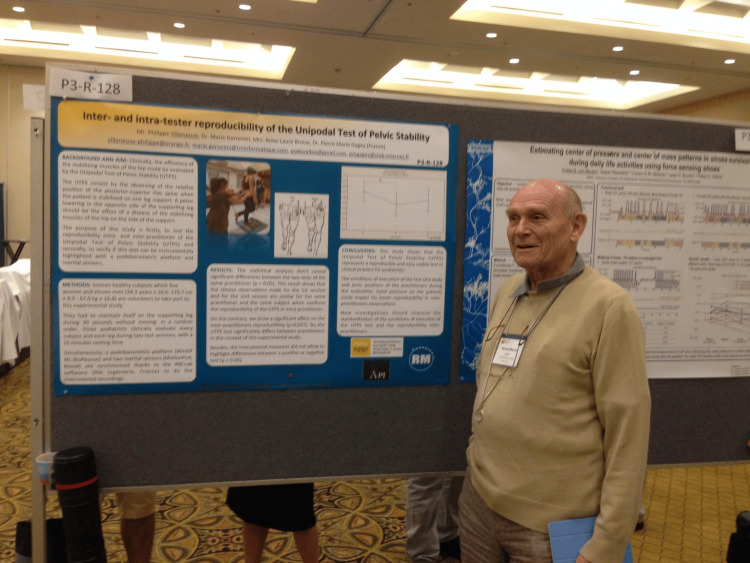
Pierre-Marie Gagey during the International Society for Postural and Gait Research (ISPGR) World Congress in Vancouver, Canada, 2014. Source: from the personal archives of P. Villeneuve (with the kind permission of the author).

Dr. Gagey passed away on February 18, 2023, and is buried at Savigny-lès-Beaunes Cemetery in the Côte-d'Or region, France (Figure 3). His legacy of kindness, humility, and extensive knowledge of human postural control will remain forever in our memories. Even at 94 years old, he retained the energy to teach seminars on stabilometry, deliver lectures, and discuss about human postural sway [76,88].

Posturology remains the discipline that analyzes the dysfunctional muscular tonus in standing and studies the appropriate postural “primary entrance” - understood as those sensors whose stimulation leads to changes in the management of the fine postural control system - to achieve health improvements. Gagey and Weber always advocated for these stimulations only when medical clinical tests failed to identify a cause for orthostatic position dysfunction [4]. In such cases, the perspective of posturology could be applied: it requires a symptomatic patient without a medically confirmed etiology, out-of-limit stabilometric parameters, asymmetrical tone of postural muscles, and an immediate change in asymmetry (or symptoms) when one or more system information inputs are manipulated. However, Gagey stated that postural diagnosis could only be made retrospectively, as a “postural treatment” should ultimately heal a “postural patient." These ideas may have been misunderstood by some clinicians who attempted to treat various musculoskeletal problems through postural-entry modifications. While this approach has been criticized [89], successful cases have been reported where different postural manipulations affected the fine postural control system [63,90-92].

Another critical issue remains the integration of peripheral information into the central nervous system, its weighting, and the role of cognition in this process. Maurer et al. proposed a multisensory interaction model in which sensory cues are weighted and re-weighted based on stimulus amplitude, frequency, and stimulus conditions [93], a model further supported by other researchers [94]. In addition, cognitive tasks impact postural control, both in healthy and impaired subjects, showing an increase in postural sway and worsening balance reactions [95,96]. This has led to criticisms of posturology due to its "bottom-up" approach, even though human postural control is inherently complex. It is unlikely that Dr. Gagey believed that postural patients could be cured solely by modifying sensory inputs in stance; rather, his goal was to improve muscle tone asymmetries, which could, in turn, alleviate the patient's symptoms. From now on, studying the fine control system of human posture must remain attentive to advances regarding the sensory integration of information, how the central nervous system manages it, and the role of various central mechanisms in maintaining the standing position.

## Conclusions

Pierre-Marie Gagey's contributions to posturology have profoundly influenced the understanding and clinical application of human postural control. His work highlighted the intricate balance maintained by the integration of peripheral and central nervous system inputs, with an emphasis on the fine-tuned adjustments made by postural muscles. Gagey's pioneering efforts in stabilometry, particularly his standardization of measurement techniques, provided a reliable framework for assessing postural balance and detecting abnormalities in the postural system.

His curiosity about the mechanisms of stabilization of the standing human being and the findings of asymmetry of postural muscle tone led this French doctor to become interested in postural control, bringing stabilometry, posturographic recording, and the various stabilometric parameters to a whole series of clinical specialties, such as otorhinolaryngology, ophthalmology, manual therapy, and podiatry in southwestern Europe. This new discipline allows us to interact with the subject through the various postural inputs, even to relieve certain musculoskeletal symptoms. From here, we must follow his enthusiasm to delve deeper into the complex mechanism of human postural control and learn to interact with it from a clinical point of view.
